# Lives of the Lonely: How Collaborative Consumption Services Can Alleviate Social Isolation

**DOI:** 10.3389/fpsyg.2022.826533

**Published:** 2022-03-07

**Authors:** Merlyn A. Griffiths, B. Yasanthi Perera, Pia A. Albinsson

**Affiliations:** ^1^Department of Marketing, Entrepreneurship, Hospitality and Tourism, University of North Carolina at Greensboro, Greensboro, NC, United States; ^2^Department of Organizational Behaviour, Human Resources, Entrepreneurship, and Ethics, Brock University, St. Catharines, ON, Canada; ^3^Department of Marketing and Supply Chain Management, Appalachian State University, Boone, NC, United States

**Keywords:** social isolation, loneliness, sharing economy, paid friendships, consumer vulnerability, collaborative consumption, community

## Introduction

Loneliness and social isolation are significant public health concerns that affect individual and community wellbeing. Certain urban centers have seen an increase in “lonely deaths” which entail “people, often elderly, dying alone without anyone noticing” (Rashid, 2017). Termed “godoska” by South Koreans, and “kodokushi” by the Japanese (Rashid, 2017), this “death by isolation” (Albinsson et al., 2021) is an extreme consequence of loneliness. Research findings indicate that individuals' health-related behaviors, their mental and physical health, as well as their risk of death are influenced by the quantity and quality of their social relationships (Umberson and Karas Montez, 2010). According to the Cacioppo Evolutionary Theory of Loneliness, in all age groups, the experience of feeling lonely elicits a host of behavioral and biological processes that contributes to premature death (National Institute on Aging, 2019). Those that are isolated or less socially integrated are physically and psychologically less healthy and thus at greater risk of mortality (Shankar et al., 2011). While this public health concern is being addressed at multiple levels (e.g., government and local community programs), another avenue of exploration is whether sharing economy (SE) initiatives can foster human connections and thereby reduce social isolation and loneliness.

## Lived Experience of Loneliness and Social Isolation

The severity of health and wellbeing implications of loneliness and social isolation has led various health and governmental bodies to acknowledge this as a social health crisis, classify the risks, and advocate for initiatives that can mitigate its effects (Klinenberg, 2016; Nyvist et al., 2016). For instance, the U.S.-based Centers for Disease Control and Prevention (CDC) has categorized loneliness as an epidemic that has been exacerbated by the COVID-19 pandemic. Loneliness is “the feeling of being alone, regardless of the amount of social contact” whereas social isolation is “a lack of social connections” (CDC.gov, 2021). More specifically, social isolation refers to the “quality and quantity of the social relationships a person has at individual, group, community and societal levels” (Scottish Government, 2018, p. 5). Generally, those living alone, having limited social connections, and sporadic social interactions are deemed as being socially isolated (Holt-Lunstad et al., 2015). For some, social isolation can lead to loneliness, whereas others can feel lonely without being socially isolated (CDC.gov, 2021). Thus, loneliness is a subjective feeling and isolation is an objective assessment of an individual's social network (Yeh, 2017). Research, however, points to the synergistic interaction between loneliness and social isolation. For example, (Beller and Wagner, 2018, p. 810), in exploring the predictive effect of loneliness and social isolation on mortality, found that “the higher the social isolation, the larger the effect of loneliness on mortality, and the higher the loneliness, the larger the effect of social isolation.” As people age, and become less ambulant and self-sufficient, their vulnerability to social isolation and loneliness increases the potential for cognitive decline, depression, weakened immunity, anxiety, obesity, and heart disease (Shankar et al., 2011; National Institute on Aging, 2019). Besides the elderly, loneliness also impacts young adults and mothers with small children (McDonald, 2021). A study of loneliness across cultures, age, and gender found that younger people are now more susceptible to loneliness and social isolation than the elderly (Barreto et al., 2021). This study also found that men are lonelier relative to women, with younger men from individualistic cultures being the most vulnerable. In a systematic review of initiatives to reduce loneliness among the elderly, Hagan et al. (2014) found that technology-based options were among the most effective. Given that technology is the key driver of collaborative coordination within the SE (Pouri and Hilty, 2021), various initiatives that mitigate loneliness exist within this domain (see Veen, 2019; Albinsson et al., 2021).

## Collaborative Platforms For Sharing

The SE embodies collaborative consumption, which involves “people coordinating the acquisition and distribution of a resource for a fee or other compensation” (Belk, 2014, p. 1597). Thus, while some SE initiatives are purely profit driven, others are more community-oriented (see Acquier et al., 2017). Besides contributing to economic well-being, it implies interactivity within and around the community allowing for the formation of temporary connections. Research suggests that sharing of goods from mundane items like sugar to valued assets like home and cars, has the potential to build social ties and strengthen community bonds (Albinsson and Perera, 2012; Veen, 2019). Indeed, some SE initiatives that involve extended contact (e.g., CouchSurfing), or those involving a community hub (e.g., tool banks, toy libraries, coworking spaces, and meal sharing) that foster social interactions may address the issues of loneliness and isolation in the process of delivering their specific services or products (Albinsson et al., 2021). However, in this opinion article, we address SE platforms that are specifically geared for the purpose of alleviating loneliness and social isolation. In doing so, we pose questions regarding the authenticity of the relationships formed between the parties involved and discuss avenues for future research.

### Community-Based Sharing for Alleviating Social Isolation and Loneliness

The community-based economy, which entails peer-to-peer sharing initiatives as opposed to business-to-consumer sharing (Acquier et al., 2017) is an understudied area of research (Lai and Ho, 2021). Community-based sharing initiatives emphasize social ties, trust, reciprocity, and shared visions of building social capital (Albinsson and Perera, 2012). In addition to monetary gain, early conceptualizations of community-based sharing initiatives highlighted social interaction, community involvement and philanthropy or altruism and sustainability-orientation (Ozanne and Ballantine, 2010; Albinsson and Perera, 2012). A more recent conceptualization frames these alternative consumption initiatives as Communities of Benefit Exchange, defined as “A community of people with a shared purpose or interest who engage in alternative consumption practices across diverse channels in the pursuit of benefits that can include monetary or non-monetary gain” (Bajaj et al., 2021, p. 1409). Many community-based economy initiatives are designed to “empower communities and serve as a vehicle for wider social change, emancipation, and solidarity” (Acquier et al., 2017, p. 6). In our discussion we highlight sharing communities, some of which may entail monetary exchanges (see Table 1). For example, *Good Gym* helps participants get fit while completing errands for the elderly, spending time with them, and engaging in other community service work, thereby strengthening community connections. *Thuisafgehaald*, a meal sharing platform, connects volunteer cooks with those who need home cooked meals. Individuals in a community spend time together which increases the potential for developing authentic connections. In contrast to these examples, other SE platforms are based on users purchasing access to people with whom to share time, space, and activities. For example, *Rent-a-Friend* offers over 600,000 friends from around the world for hire for a range of activities from accompanying one to an event, connecting on hobbies and recreational interests, to virtual friendships through Facetime and Zoom. *Rent-a-Cyber-Friend* offers online and digital interactions through Facebook, Messenger, Skype, WhatsApp, and other chat-based platforms. *FriendPC* adds a different twist with a “virtual girlfriend” option. While sites like these advocates digital communication, there is nothing to deter users and friends from meeting face-to-face. Those seeking more intimate connections turn to sites like *Cuddle Comfort*, that match snugglers (those available for pay) with “snugglees” who purchase the time of someone to snuggle up with, or what the site calls “safe spooning.” Snugglees include those who are lonely and socially isolated and those who want temporary comfort without the pressures of a committed relationship. As indicated by these examples, SE platforms can be harnessed and directed toward populations that may be isolated or lonely. However, the potential outcome is dependent on individuals' desire to improve the state of their physical and emotional wellbeing.

**Table 1:** Examples of sharing initiatives addressing social isolation and loneliness.

**Sharing site**	**Offerings**	**Users and members**	**Connections**	**Cost to user**
Rent a Friend www.rentafriend.com	Users hire a friend, strictly platonic friendship site.	621,585 friends available for hire worldwide as of January 17, 2022	In person connections or virtual—Facetime, Zoom, Texting, Phone.	Rentable friends can earn up to $50 per hour.
Cuddle Comfort www.cuddlecomfort.com	Cuddle Comfort is a community of people that love to cuddle. We help you create friendships based on cuddling that are pressure free and with no expectation of something more.	230,000 global users. 1,200 professional cuddlers across 50 states in the U.S.	Snuggle buddies meet In-person	Members pay for a professional cuddler. Free to join. Professional snugglers earn $80–400 per night.
Rent a Cyber Friend www.rentacyberfriend.com	Similar concept of pen pals. Services are posted in the form of gigs—e.g., help a tourist in a city, help someone practice speaking a language, or socializing with someone when they are lonely.	No information given	Virtual communications	Membership required. Payment is per minute with a 20% platform fee.
Rent a Local Friend www.rentalocalfriend.com	Connects travelers with the local people of an area as a tour guide, chef, photographer.	No information given	In-person and online chats	No information given.
Papa www.papa.com	Family on demand. Papa (older adults) and Papa-pals (younger adults) get matched for companionship, conversation, watch movies, house tasks, meal prep, errands, transportation, and more.	Available in most U.S. states	In-person	Papa must have health plan membership. Papa-pals earn up to $15 per hour. Up to $3,000 per month.
FriendPC www.friendpc.com	Companionship as a virtual sugar baby, life coach, virtual gamer, virtual friend, and virtual girlfriend services.	No information given	Virtual, phone, text, video or in-person	Site is paid 5% of the earnings.
Rent A Sister (Japan based)	Companionship for “Hikikomori” young men who have withdrawn from society and refuse to leave their bedrooms.	No information given	No information given	No information given.
Fiverr www.fiverr.com	Freelancer marketplace. Services offered includes web design, content creation, logo design, and friendship.	No information given	Phone, video, 24-hour chat	$5–50 per hour.
Good Gym www.goodgym.org (United Kingdom)	A community of runners, walkers and cyclists, who get fit by doing “good”—group runs, community missions, coach visits (elder person with life experiences of interest), and missions.	50 areas in the U.K.	In-person	Monthly donations accepted. No membership fees.
Thuisafgehaald https://thuisgekookt.nl/ (Netherlands)	Matching volunteer neighborhood home cooks with those needing meals, regularly or on occasion. Users can share, eat meals and chat.	10 cities in the Netherlands 16,500 home cooks registered 100,000+ meals shared annually	In-person	Non-profit.

### Fallacy of Paid Friendships

While numerous SE initiatives aim to lessen loneliness and social isolation, the growing body of research on SE highlights its complex and paradoxical nature (e.g., Acquier et al., 2017), which includes the possibility of unforeseen negative externalities (Griffiths et al., 2019). Typically, friendships are formed over time and entail involved parties developing a mutual sense of trust and comfort. Therein lies the fallacy imparted by pay-for-friendship based SE entities that are positioned as authentic spaces for social connections. In such settings, participants' safety is solely based on their ability to self-protect and guard against revealing personal information that may be used to commit fraud. Those meeting face-to-face must be conscious of their surroundings, location of the meet-up, and ensure that they are not followed after the meet-up has ended. The question of malevolent intent is one that must be considered in the context of understanding the feasibility and authenticity of these friendships. SE initiatives that are more capitalistic in nature are rooted in self-interest and the pursuit of profit, thereby having the potential to produce negative effects (Griffiths et al., 2019). By relying on price-based exchange systems to coordinate between friends and friend-seekers they often depersonalize exchanges (Escobedo et al., 2021). SE-based initiatives that involve the renting of oneself as a companion or friend makes this even more evident.

## Moving Forward

To date, little research has explored SE initiatives that purport to address loneliness and social isolation. We call for researchers to devote more attention to examining such entities to assess their efficacy and implications. For instance, SE strategies that address quality of life for various genders and age groups should also be explored. Additionally, there is a need to examine providers' motivations to offer their services (e.g., altruistic, self-serving). Additionally, as some users may have physical or mental ailments, how do platforms ensure the wellbeing of a user who may be in crisis? To what extent are the platforms vetting their service providers? While platforms like Papa.com indicate that they offer training to their service providers, to what extent does this entail formal certifications under health care regulations? Researchers should also explore the dynamics between various stakeholders (e.g., volunteers, clients, consumers, social entrepreneurs, and among others) and how these can foster wellbeing. For instance, examining the dynamics of the relationship between the users, their families, and the providers (e.g., as in the case of “Sisters for Hire” in Japan) could provide more clarity on aspects that support or detract from meeting the initiatives' objective of alleviating social isolation and loneliness. Furthermore, it behooves researchers to examine the broader societal implications of these services normalizing companionship for hire. For instance, does this result in objectification of service providers? In this regard, recent discussions of the availability of sex dolls (mostly female) revolves around whether such engagement can lead to the objectification of women (Belk, 2018). From a wellbeing perspective, user and provider protection, as well as whether such services alleviate loneliness and isolation are avenues for future research. Essentially, it is necessary to consider whether these SE platform services connect people with the wider community and improve wellbeing in the long term or whether they are SE-based marketplace offerings profiting from consumers' vulnerabilities.

## Author Contributions

All authors listed have made a substantial, direct, and intellectual contribution to the work and approved it for publication.

## Conflict of Interest

The authors declare that the research was conducted in the absence of any commercial or financial relationships that could be construed as a potential conflict of interest.

## Publisher's Note

All claims expressed in this article are solely those of the authors and do not necessarily represent those of their affiliated organizations, or those of the publisher, the editors and the reviewers. Any product that may be evaluated in this article, or claim that may be made by its manufacturer, is not guaranteed or endorsed by the publisher.
